# Digitale Tools in der Fort- und Weiterbildung im Rahmen eines Digital-Media-Konzepts

**DOI:** 10.1007/s00101-024-01466-6

**Published:** 2024-10-11

**Authors:** Ulrike Schlüter, Ralf Sowa, Ingmar Finkenzeller, Thomas Mencke, Daniel A. Reuter

**Affiliations:** 1 Klinik und Poliklinik für Anästhesiologie, Intensivmedizin und Schmerztherapie, Universitätsmedizin Rostock, Schillingallee 35, 18057 Rostock, Deutschland; 2 Klinik für Anästhesiologie, Universitätsmedizin Göttingen, Göttingen, Deutschland

**Keywords:** Ausbildung, Digitales Lernen, Asynchrones Lernen, Lebenslanges Lernen, FOAM, FOAMed, Education, Digital learning, Asynchronous learning, Lifelong learning, FOAM, FOAMed

## Abstract

Heutzutage gibt es viele Online-Angebote zu Aus- und Weiterbildung in der Anästhesiologie, Intensivtherapie, Schmerz‑, Notfall- und Palliativmedizin. Von traditionellen Lehrbüchern und Präsenzveranstaltungen über Lernplattformen, Applikationen und Podcasts bis zum Training im Simulationszentrum und Virtual-Reality-Szenarien; es gibt viele Wege, sich fort- und weiterzubilden. Insbesondere durch die Coronapandemie gab es große Fortschritte, medizinische Lerninhalte besser zugänglich zu gestalten, um u. a. auch die Übertragung von Wissen zu beschleunigen.

Um Kollegen auf das Ziel des Life-long Learning mitzunehmen und alle modernen Tools miteinzubeziehen, empfehlen wir die Entwicklung eines Digital-Media-Konzepts, welches auf jede anästhesiologische Abteilung zugeschnitten ist. Zunächst gilt es, die Ziele einer Abteilung zu erfassen, z. B. ob vorhandene Lehr- und Lernmaterialien digital zugänglicher gemacht werden können, im Sinne des asynchronen Lernens. Danach sollten die Ressourcen dieser Abteilung erfasst werden, z. B. welche Lernplattform schon genutzt wird, oder ob/wie Social Media eine Rolle spielen soll. Eine oder mehrere verantwortliche Personen sollten dann für die Erhaltung des Konzepts bestimmt werden. In diesem Zuge empfiehlt es sich, einheitliche Qualitätskriterien, mit denen digitale Inhalte überprüft werden, zu entwickeln.

Durch die Unterstützung der eigenen Abteilung kann konventionelle Fort- und Weiterbildung mit neuen digitalen Möglichkeiten gut kombiniert werden. Hiermit können speziell individuelle Dienstmodelle, wechselnde Beteiligung an Präsenzveranstaltungen und verschiedene Lerntypen berücksichtigt werden. Digitale Tools sind vielfältig, stellen eine großartige Bereicherung für die Aus- und Weiterbildung jedes Teammitgliedes einer anästhesiologischen Abteilung dar und werden uns in die Zukunft begleiten.

## Abstract (en)

There are currently many online resources for medical education during residency and beyond in anesthesiology, intensive care, pain, emergency and palliative medicine. From traditional textbooks and in-person events to learning platforms, apps, podcasts, simulation training and even virtual reality, there are many ways to supplement traditional residency curricula and continuing medical education. The coronavirus disease 2019 (COVID-19) pandemic has been instrumental in making medical education content more accessible and, among other things, accelerate the transfer of knowledge.

To include all colleagues in the goal of life-long learning using these modern tools, we recommend the development of a digital media concept that is individually tailored to each department of anesthesiology. First, the goals of the department should be defined, e.g., can existing teaching materials be made more digitally accessible for asynchronous learning? Then, department resources should be compiled, e.g., what learning platforms are already being used and if and how social media should play a role? One or more persons should be named responsible and maintain the new concept. In this context, it is essential to develop quality criteria to properly assess the digital content.

With the support of the department, conventional teaching methods can be combined with new digital possibilities in residency education and beyond. In this way, individual shift models, various levels of participation in live teaching events and different types of learners can be taken into account. These diverse digital tools can enrich the training and further education of every team member in an anesthesiology department and will accompany us well into the future.

## Einführung

Digitale Medien: Wie geht man als kürzlich approbierter Arzt damit um? Wie nähert man sich als Chef? Hierbei gilt es, zwei unterschiedliche digitale Erfahrungsgrade zusammenzubringen. Es ist eine praktische Herausforderung, mit den erfahrensten Kollegen sowie den jüngsten Ärzten in Weiterbildung gemeinsam in die Welt der digitalen Medien einzutauchen.

In diesem Artikel wollen wir Sie durch eine Auswahl der digitalen Möglichkeiten sowie deren Chancen und Risiken zur Unterstützung der Fort- und Weiterbildung leiten. Der Fokus liegt hierbei auf der Zugänglichkeit und der leichten Integration in den Weiterbildungsalltag. Die Vorstellung von derartigen Werkzeugen erfolgt im Rahmen eines übergeordneten Digital-Media-Konzepts, anwendbar auf den eigenen Standort.

## Hintergrund

Ein gewandter Chef, aktuell meist Generation X, kennt sich sicherlich mit einigen modernen Online-Tools aus, ist aber in aller Regel selbst mit traditioneller Lehre, inklusive frontalem Unterricht und klassisch analogen Lehrbüchern, groß geworden und entsprechend vertraut. Der neue Arzt in Weiterbildung ist eher Generation Y und Z, „digital native“, hat in der Regel gerade sein Studium abgeschlossen und entsprechend kürzlich noch viele Berührungspunkte mit Online-Lehre gehabt. Einige Kollegen brennen für Wissensvermittlung via Zoom und allgemein Social Media, wohingegen andere mit den Begriffen TikTok und Podcast nichts anfangen können. Wie ermöglicht eine Klinik für Anästhesiologie einen strukturierten Zugang zu digitalen Medien, der den Bedürfnissen der eigenen Kollegen angepasst ist?

Von Podcasts über YouTube-Tutorials bis Ultraschalllernprogrammen – heutzutage wird man von modernen Online-Lehrangeboten überhäuft. Die Veränderungen in der digitalen Welt sind vergleichbar mit der Entwicklung des Buchdruckes im 15. Jh. Informationen können schneller und weiter verbreitet werden als je zuvor. Auch die Weiterbildung in der Anästhesiologie, Intensiv‑, Schmerz- und Palliativmedizin sowie Notfallmedizin passt sich diesen Neuerungen an und kann allen Kollegen eine wertvolle Erweiterung der traditionellen Lehrmethoden bieten.

Die digitale Wissensvermittlung ist einer der beliebtesten und sich am schnellsten entwickelnden Bereiche in der Aus- und Weiterbildung von Fachpersonal im Gesundheitswesen [1]. Alle Generationen an Ärzten können von neuen Herangehensweisen der Lehre profitieren [2]. Verschiedene Lerntypen und auch diverse Arbeitszeitmodelle und Lebensstile können besser berücksichtigt werden. Ein wichtiges Stichwort hierbei ist das asynchrone Lernen, welches sich dadurch auszeichnet, dass Lernende zu einem selbstgewählten Zeitpunkt Inhalte konsumieren können.

Spätestens seit Beginn der Coronapandemie 2020 ist deutlich geworden, dass Online-Angebote für Weiterbildung, Fortbildung und Lehre ausgebaut werden müssen, um alle Kollegen bei der multimodalen Wissensvermittlung in möglichst allen Lebenslagen mitnehmen zu können. Dieser Aspekt ist absolut ein Teil des Zieles des „Life-long Learning“ (lebenslanges Lernen). Dieses Life-long Learning [3], in der Medizin auch speziell Continuing Medical Education (Fortbildungen) genannt, ist grundlegend wichtig, um kontinuierlich auf dem sich stetig weiterentwickelnden, aktuellen Stand der fachrelevanten, wissenschaftlichen Erkenntnisse und Behandlungsansätze zu bleiben.

Die alltäglichen beruflichen Herausforderungen als Arzt in Weiterbildung sind allein schon beachtenswert; insbesondere in der Anästhesiologie und Intensivmedizin kam es durch tiefgreifende Veränderungen im Gesundheitswesen innerhalb der letzten 20 Jahre zu einer kontinuierlichen Verdichtung der Arbeitsprozesse im OP und auf den Intensivstationen. Das Risiko für Burn-out ist insbesondere für Ärzte in der Weiterbildung Anästhesiologie sehr hoch. Eine Studie des Royal College of Anaesthesists 2017 zeigte, dass 61 % der Residents empfanden, dass ihr Beruf ihre mentale Gesundheit negativ beeinflusst hatte, während 85 % ein höheres Risiko für Arbeitsplatz-Burn-out aufwiesen [4]. Als Arzt in Weiterbildung hat man die Hauptaufgabe, sich fachrelevantes Wissen anzueignen und unter Anleitung praktisch anzuwenden, um später die Facharztanerkennung zu erwerben. Somit bietet sich für eine Klinik die große Möglichkeit, durch eine vielfältige und bedarfsorientierte Unterstützung dieser Lernprozesse aktiv zur Reduktion des Stresslevels und zur Erhöhung der Zufriedenheit in unserem Beruf beizutragen [4, 5].

In einigen Weiterbildungsstätten werden digitale Tools bisher noch nicht oder nur in sehr geringem Ausmaß genutzt, wohingegen in anderen digitale Lernplattformen, Online-Bibliotheken und digitale Kommunikationskanäle vielfältig zur Verfügung stehen. Häufig sind Information über fachspezifische Inhalte eher heterogen. Hier kann ein Rahmenkonzept mit explizitem Raum für digitale Tools helfen, sodass der Nutzen dieser neuen Werkzeuge für alle, sowohl die Ärzte in Weiterbildung wie auch für die gesamte Klinik, am größten ist.

Im ersten Teil dieses Artikels wollen wir die Bedeutung eines prinzipiellen Konzepts zur Integration von digitalen Medien für eine klinische Abteilung diskutieren. Im zweiten Teil werden spezielle Beispiele, wie digitale Tools in der Fort- und Weiterbildung verwendet werden können, beschrieben.

## Entwicklung eines Digital-Media-Konzepts

### Grundlagen: Ziele und Ressourcen

Zahlreiche digitale Tools werden in der Weiterbildung bereits spontan genutzt und digitale Inhalte geteilt. Dies können kommunikative, organisatorische Kanäle wie z. B. WhatsApp oder Signalgruppen zur Erfassung von Dienstwünschen oder wissensvermittelnde Inhalte wie Podcast-Beiträge oder Smartphone-Applikationen (Apps) sein. Informelle Kanäle zum Austausch von Wissensinhalten im Team wird es stets geben. Zu dem Ziel, dass möglichst alle Kollegen, insbesondere alle Ärzte in Weiterbildung, dies strukturiert nutzen können, bedarf es genau einer solchen, übergeordneten Struktur. Nicht alle Tools und deren Online-Inhalte sind qualitativ gleichwertig und praktikabel. Im Folgenden stellen wir ein beispielhaftes, übergeordnetes Digital-Media-Konzept, welches für die Einordnung dieser Aspekte ein sinnvoller Ausgangspunkt sein kann, vor. Zunächst sollten die Rahmenbedingungen eines derartigen Konzepts in der einzelnen Institution erfasst werden (Infobox 1).

#### Infobox 1: Rahmenbedingungen der Abteilung für ein Digital-Media-Konzept erfassen

- Wie können digitale Inhalte in die Weiterbildung sowie Fortbildungen integriert werden?
- Wie kann die Organisation der Weiterbildung digital unterstützt werden?
- Welchen Bedarf der digitalen Aufbereitung der vorhandenen Materialien gibt es? Zum Beispiel Hochladen von Fortbildungen, Erstellung von einem Podcast, App-basierte Inhalte
- Soll eine Präsenz auf Social Media etabliert werden? Wenn ja, welche Kanäle und welche konkreten Ziele sollen auf jedem Kanal verfolgt werden?
- Welche Zeitinvestition ist notwendig/möglich?
- Welche finanzielle Investition ist notwendig/möglich?

#### Formale Eckpunkte

Wenn Inhalte auf Social Media präsentiert werden sollen, bedarf es zunächst einer Abstimmung auch der formalen Eckpunkte mit der Krankenhausleitung. Selbstverständlich müssen gängige professionelle Standards befolgt und die Kultur der Social-Media-Kanäle beachtet werden [6]. Inhalte sollten im Einklang mit der medizinischen Einrichtung als Ganzes sein. Hierzu ist ein enger Kontakt mit der Presse/Medienabteilung empfehlenswert. Welche Kanäle genau genutzt werden, muss individuell abgewogen werden. In Rostock z. B. ist die Klinik für Anästhesiologie, Intensivmedizin und Schmerztherapie (KAIS) auf Instagram aktiv; aus Göttingen findet man den Young-Urban-Anesthesiologists-Podcast auf deren Homepage und weitere Inhalte auf X (ehem. Twitter).

#### Vorhandene Ressourcen evaluieren

Im zweiten Schritt sollte eine Erfassung der vorhandenen Ressourcen stattfinden: Welche Tools nutzen die Lehrbeauftragten der Klinik, außerdem die Fach- und Oberärzte in der Aus- und Weiterbildung? Über welche Softwarelizenzen verfügt die Klinik/Abteilung bereits? Gibt es eine Verbindung zu einer medizinischen Fakultät mit weiteren digitalen Mitteln für die Hochschuldozenten? Welche Kanäle und Ressourcen nutzen die Ärzte in Weiterbildung schon? In welcher Form sind klinikinterne Inhalte schon „online …“ und asynchron verfügbar etc. (Infobox 2)? Feedback und Ideen aus dem Team sollten niederschwellig integriert werden.

##### Infobox 2: Erfassung von Ressourcen der Klinik/Abteilung

- Wie ist das Nutzungsverhalten von Lehrenden und Lernenden?
- Interne Statistik zu beliebten Kanälen und Quellen erstellen
- Austausch von Erfahrungen zur eigenen Lehre bzw. zu Erfahrungen einer nahegelegenen medizinischen Fakultät
- Fragen u. a. an die IT-Abteilung zu: Hardware, Software, Lizenzen, Datensicherheit, Vernetzung: z. B:. Gibt es eine Kooperation mit einer Universitätsbibliothek für den Zugriff auf wissenschaftliche Zeitschriften und Lehrbücher?
- Wird eine Lernplattform genutzt?
- Welche wiederkehrenden technische Schulungen gibt es? Können diese ausgebaut und für neue Medien erweitert werden?
- Stand der Homepage erfassen

Zu den allgemeinen Ressourcen zählt insbesondere die IT-Abteilung mit Hardware, Software, Vorgaben zu Datensicherheit, Serverplatz sowie Bereitstellung von Zugängen außerhalb der Klinik. Die Schulung des Personals u. a. der Ärzte, des Pflegeteams, der Psychologen und Mitarbeiter des Sozialdienstes zählt auch zu Ressourcen – allen sollte der Zugang zu digitalen Medien ermöglicht werden. Die „Last,“ Kollegen in technischen Fragen zu unterstützen, sollte nicht auf wenigen Einzelpersonen ruhen, die sich aus Eigeninitiative damit beschäftigen. Eine adäquate Anzahl an Arbeitsplätzen (Tablets, Laptops etc.) muss bereitgestellt werden. Auch die digitale Aufbereitung von bereits vorhandenen Materialien bedarf Zeit und Anpassung an die verfügbaren Strukturen. Eine zeitgemäße Überarbeitung von bestehenden Dokumenten mit klickbaren Links, neuen Grafiken und interaktivem Interface ist wichtig. Eigene Forschung in der Lehre, z. B. zur Effektivität von verschiedenen Hybridlernmethoden, helfen enorm, den eigenen Studierenden sowie den Ärzten in Weiterbildung ein wissenschaftsbasiertes Curriculum anzubieten [7, 8].

### Aufbau: Verantwortlichkeiten und Begleitung

Im weiteren Schritt des Aufbaus eines Digital-Media-Konzepts bedarf es der Benennung einer oder mehreren verantwortlichen Personen (Infobox 3).

#### Infobox 3: Verantwortliche identifizieren und Arbeitsfähigkeit herstellen

- Eigenschaften: Eigeninitiative, Erfahrung in diversen Social-Media-Kanälen, inkl. Netiquette, exzellente professionelle Kommunikation, sehr gute Soft Skills und interprofessionelle Vernetzung, u. a. mit der Pressestelle
- Plus: Erfahrung in der Erstellung von Online-Inhalten, in der Nutzung diverser Design- und Grafikprogramme, sehr gute Englischkenntnisse
- Zeitaufwand definieren und bereitstellen
- Zusammenarbeit zwischen Lehrenden und Lernenden vermitteln
- Arbeitsplatz mit adäquater Technik bereitstellen
- Verantwortliche Person in ihrer Arbeit mit anderen Abteilungen unterstützen
- Rückmeldungskanäle zur Messung der Wirksamkeit der Arbeit etablieren
- Teilbereiche definieren und delegieren: Fortbildungsinhalte erstellen, digitaler Zugang, Social Media, Homepage etc.

Die Erfahrung der letzten 4 Jahre aus Rostock zeigt, dass der Aufbau eines Digital-Media-Konzepts sehr zeitaufwendig ist und „nebenbei“ nicht zu bewältigen ist. Der verantwortlichen Person müssen zeitliche Ressourcen für ein solches Projekt zur Verfügung stehen; außerdem bedarf es viel Enthusiasmus und Engagement, ein solches System zu etablieren. Dabei wird der erforderliche zeitliche Aufwand anfangs häufig unterschätzt.

Auch in Göttingen wurde die Entwicklung eines solchen Teilbereichs mit der Benennung einer verantwortlichen Person für die Leitung des Bereiches Digitale Medien in der Klinik unterstützt. Die Aufgabe dieser bereichsverantwortlichen Person liegt in der Bewertung, Distribution und letztendlich in der Erzeugung von digitalen Inhalten [9]. Die Besetzung aus dem (fach-)ärztlichen Personal ist nahliegend, um digitale Medien und Inhalte der gleichen Qualitätskontrolle unterlaufen zu lassen wie „traditionellen“ Printmedien. Ebenfalls bietet sich hier ein generationenübergreifender Ansatz an, der die Erfahrung und Expertise des Stammpersonals mit den Kenntnissen und Bedürfnissen der Berufseinsteigern im Umgang mit den aktuellen digitalen Plattformen aus dem Studium oder dem Privatleben verbindet.

Die Klinik für Anästhesiologie der Universitätsmedizin Göttingen hat einen solchen Verantwortungsbereich „Kommunikation und neue Medien“ geschaffen und Freiräume für die Ausgestaltung eingeräumt. Die Pflege der abteilungsspezifischen Outlets in den sozialen Medien ist hier der zentrale Tätigkeitsbereich. Hervorzuheben ist der Podcast „Young Urban Anesthesiologists“ (YUAN) der Abteilung, der überregional sehr gut angenommen wird und von der DGAI mit dem Thieme Teaching Award ausgezeichnet wurde [10]. Die Erstellung dieses Podcasts – und das gilt generell für die Erstellung von Podcasts – bedarf eines großen Aufwands an Zeit sowie des Vorhandenseins entsprechender technischer Ausrüstung [11, 12]. Am Beispiel Göttingen kann man sehen, wie ein solches Projekt bzw. Produkt zu einem Aushängeschild der Institution werden kann; andererseits impliziert dies nicht, dass jede Klinik/Abteilung eigene Podcasts generieren sollte und muss.

Das Selbstverständnis des Bereichs ist das eines ärztlichen kreativen „Content Creator“: Dieser hat zwar eine hohe technische Affinität, ist aber nicht, wie die Krankenhaus-IT-Abteilung, Ansprechpartner für primär technische Aufgaben. Vielmehr kann er neue Inhalte, wie Apps, identifizieren und bewerten [13]. Neue Angebote können somit schnell erkannt und die Sinnhaftigkeit für die eigenen Kollegen geprüft werden [14]. Die Position stellt somit einen Netzwerkknotenpunkt für die jeweilige Klinik dar [15].

Ein Digital-Media-Konzept darf nicht als starre Einheit verstanden werden; neue relevante digitale Themen aus primär anderen Bereichen, wie z. B. die Einführung des eLogbuchs in der Weiterbildung, sollten mitintegriert werden. Die langfristige Begleitung und Anpassung des Konzepts sollten stets präsent sein (Infobox 4). Das praktische Maß der Umsetzung eines Digital-Media-Konzepts bestimmt jede Abteilung anhand seiner Bedarfsanalyse.

#### Infobox 4: Begleitung des Digital-Media-Konzepts

- Generationsübergreifend, interprofessionell
- Regelmäßige Erstellung und Begutachtung der Inhalte
- Vernetzungen innerhalb eigener Abteilung
- Synergie: Personalakquise
- Anpassung von vorhandenen Medien – Apps, Lernplattform

### Speziell: Datensicherheit und Kommunikation

Ein großes Thema bei der digitalen Verbreitung von Wissen ist die Datensicherheit und Wahrung von Urheberrechten. Bei der Lernplattform studIP (s. Abschn. „Lernplattformen als Basis für digitale Wissensvermittlung“) sind Angaben zum Urheberrecht beim Hochladen von Dateien wie Vorlesungsfolien zu machen, sodass gemäß dem am 01.03.2018 in Kraft getretenen Urheberrechts-Wissensgesellschafts-Gesetz Materialien zur Verfügung gestellt werden können. Dieses Gesetz regelt, welche urheberrechtlichen Nutzungshandlungen im Bereich Bildung und Wissenschaft erlaubt sind, ohne dass es einer Zustimmung der Urheber bedarf [16]. Auch bei dem Erstellen von abteilungsinternen oder für die Öffentlichkeit gedachten digitalen Inhalten sollten Urheberrechtsfragen immer geklärt und klar markiert sein. Ein digitaler Inhalt mit Creative-Commons-Lizenz z. B. kann u. U. freier verteilt werden.

Des Weiteren wird sich oft außerhalb konventioneller geschützter Bereiche (Intranet, Telefon, E‑Mail, Vorlesung, Seminare) ausgetauscht. Hier sollte jede Abteilung klären, welche Kommunikationskanäle für welche Inhalte vorrangig genutzt werden sollten. Einzelne Bewertungen der Apps bezüglich Datenschutzthemen gehen über den Rahmen dieses Artikels hinaus. In Infobox 5 wird das beispielhafte Vorgehen zum Erstellen eines Digital-Media-Konzepts zusammengefasst.

### Zwischenfazit

Digitale Themen haben in der Weiter- und Fortbildung AINS bereits einen sehr wichtigen Stellenwert eingenommen, der in Zukunft noch weiterwachsen wird. Ein professionell verankertes Digital-Media-Konzept hilft, den raschen Wandel der Bedürfnisse und Inhalte abzubilden. Die Umsetzung eines solchen Konzepts kann neben einer verbesserten Wissensvermittlung auch signifikant zu Steigerung der Attraktivität der Klinik und Mitarbeiterzufriedenheit beitragen.

#### Infobox 5: Leitfaden zum Erstellen eines Digital-Media-Konzepts

1. Ziele erfassen
  Wie sollen digitale Inhalte in die Aus- und Weiterbildung integriert werden?
  Welchen Bedarf der digitalen Aufbereitung gibt es?
2. Erfassung von Ressourcen
  Wie ist das Nutzungsverhalten von Lehrenden und Lernenden?
  Fragen u. a. an die IT-Abteilung
  Stand der Homepage erfassen
3. Verantwortliche einbinden
  Zeitaufwand definieren und bereitstellen
  Zusammenarbeit mit Lehrenden und Lernenden vermitteln
  Arbeitsplatz mit adäquater Technik bereitstellen
4. Begleitung des Konzepts
  Generationsübergreifend und interprofessionell
  Vernetzung in eigener Abteilung

## AINS-(Weiter‑)Bildung: digital unterstützt und ergänzt

Im Folgenden wollen wir an konkreten Beispielen aus zwei universitären Standorten in Deutschland darstellen, wie Aus‑, Fort- und Weiterbildung mit konkreten digitalen Modulen unterstützt und weiterentwickelt werden können.

### Vernetzung von Informationen

Jede anästhesiologische Weiterbildungsstätte versucht, eine breite Palette an Ressourcen für Ärzte in Weiterbildung sowie Fach- und Oberärzte anzubieten. In vielen Kliniken/Abteilungen gibt es wiederkehrende Fortbildungen für alle Kollegen sowie kleinere spezielle Veranstaltungen und interne Curricula nur für Ärzte in Weiterbildung. Zum einen sind regelmäßige Weiterbildungsveranstaltungen erforderlich; zum anderen müssen Fachärzte in einem Zeitraum von 5 Jahren Fortbildungspunkte sammeln.

Zuallererst ist ein strukturierter Informationsfluss über diese diversen Angebote notwendig. Es bietet sich an, Informationen zu Weiterbildungsveranstaltungen und anderen Angeboten zusätzlich zu den traditionellen Kanälen (Flyer, Aushang, Informations-Mail) an einem zentralen digitalen Standort, z. B. auf einer Lernplattform oder auf der Homepage der Abteilung zu hinterlegen. Wichtig ist hier die engmaschige Pflege/Aktualisierung dieses Mediums. Außerdem kann ein moderner, interaktiver Newsletter mit automatischen Aktualisierungen, der Verlinkungen auf den zentralen digitalen Standort beinhaltet, auf aktuelle Informationen hinweisen.

Auch weitere Beispiele unterstreichen, wie kleine digitale Verbesserungen den Informationsaustausch effektiver gestalten: Seit nunmehr 2 Jahren wird in Rostock die tägliche Frühbesprechung, in der neben aktuellen klinischen Angelegenheiten auch der Informationsfluss über stattfindende Fort- und Weiterbildungsveranstaltungen gestärkt wird, als Live-Stream im geschützten Netzwerk übertragen. Somit ist gewährleistet, dass auch alle Kollegen an peripheren Außenstandorten und auf den Intensivstationen miteingebunden sind. Diese Live-Stream-Angebote lassen sich natürlich auch auf jegliche Form an interdisziplinären und -professionellen Fortbildungsveranstaltungen, inkl. externen Gästen, in Hybridform – mit Präsenz- und Online-Anteilen – ausdehnen.

### Lernplattformen als Basis für digitale Wissensvermittlung

Eine solide Basis für die Organisation von digitalen Angeboten bietet die abteilungsinterne Nutzung von Lern- und Austauschplattformen wie die großen Community-gestützten Open-Source-Bildungsplattformen Ilias, studIP und Moodle oder das Lernmanagementsystem Blackboard. Hier können verschiedene Module nach den Ansprüchen der übergeordneten Klinikstruktur mit Möglichkeiten der individuellen Anpassung (z. B. gestaffelte Zugriffskontrollen) konfiguriert werden. Die medizinische Fakultät der Universität Rostock nutzt z. B. studIP und Ilias. Hier werden u. a. das digitale Material der Vorlesungen und Übungsinhalte veröffentlicht sowie Vorlesungen über die Meeting Software Big Blue Button gehalten.

Auch die KAIS der Universitätsmedizin Rostock nutzt diese Plattform. Entsprechende Zugänge werden automatisch bei Eintritt eines neuen Mitarbeiters durch die IT-Abteilung erteilt. Hier werden u. a. die Frühbesprechungen gestreamt, Regelfortbildungen gehalten, wiederkehrende Pflichtunterweisungen durchgeführt, entsprechendes Material geteilt und auch der Unterricht der Studierenden koordiniert. Fort- und Weiterbildungsangebote können hierdurch teilweise orts- und zeitungebunden angeboten werden. Unsere Erfahrung ist, dass wir mit diesen asynchronen Angeboten regelhaft mehr Kollegen erreichen als mit strikten Präsenzveranstaltungen zu festen Terminen.

**Abb. 1:**
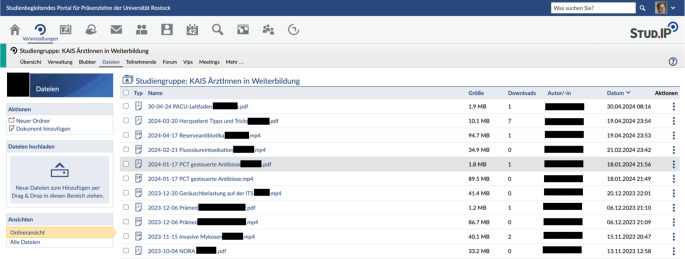
Dateien im Zusammenhang mit Veranstaltungen für Ärzte in Weiterbildung

In Abb. 1 sieht man einen Auszug aus dem Online-Ablageort für Dateien der Fortbildungen für Ärzte in Weiterbildung. Nicht alle Veranstaltungen konnten aufgezeichnet werden.

Jede Online-Lernplattform hat Vor- und Nachteile, welche jede Abteilung für sich abwägen muss. Vielerorts können Elemente einer vorhandenen Plattform genutzt werden; kleinere Abteilungen können Teilfunktionen dieser Plattformen erwerben. Des Weiteren gibt es andere Daten/Lernmanagement- und Kommunikationssysteme, die den Austausch im Team erleichtern können. Aber auch hier gilt, dass der Umgang mit diesen Online-Systemen für alle Mitarbeiter gezielt geschult werden sollte.

### Digitale Fort- und Weiterbildung: interaktiv und hybrid

**Abb. 2:**
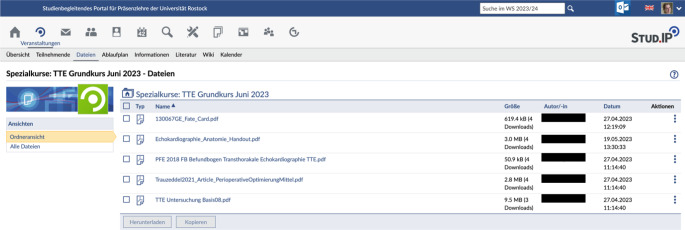
Online-Ressourcen für den TTE-Grundkurs

Digitale Fort- und Weiterbildungsangebote können auch interaktiv gestaltet werden. z. B. als Online-Diskussionsrunde mit Fragen an die Teilnehmenden und Nutzung von webbasierten Live-Feedback-Systemen wie Tweedback. Dies sind Werkzeuge, die sich bei der studentischen digitalen Lehre als sehr beliebt und effektiv herausgestellt haben [17, 18]. Auch das Konzept des „flipped classroom“, bei dem Inhalte zu Hause erarbeitet und dann während eines Seminars oder einer Fortbildungsveranstaltung besprochen werden, können mitintegriert werden. Hiermit können unterschiedliches Vorwissen, unterschiedliche Lerngeschwindigkeiten und Tagesabläufe berücksichtigt werden. In der Präsenzveranstaltung ist dann eine tiefere Diskussion möglich [19]. Dieses Konzept wird in der KAIS der Universitätsmedizin Rostock z. B. für ein fest etabliertes Kleingruppenseminar „transthorakale Echokardiographie für Ärzte in fortgeschrittener Weiterbildung“ via StudIP praktiziert. Relevante Ressourcen werden vorab an die Teilnehmer via StudIP verteilt (Abb. 2, 4 Teilnehmer), um diese dann im Kurs zu vertiefen. Diese Vorgehensweise, dass Online-Inhalte im Voraus sorgfältig geprüft und verteilt werden, entspricht dem oben genannten Digital-Media-Konzept.

Auch weiterführende digitale Technologien, wie Technology-enhanced Learning, Virtual Reality Training, spielbasiertes Lernen, insbesondere bei praktischen Fächern, wurden bereits mancherorts nicht nur in die studentische Lehre, sondern auch in die ärztliche Weiterbildung implementiert [20–22]. Außerdem können bewährte Lehrkonzepte aus dem Medizinstudium, wie Team-based Learning und Problem-based Learning digital ergänzt und in der Weiterbildung eingesetzt werden [23–25].

### Weiterbildungsinhalte digital erfassen

Die Erfassung der absolvierten Weiterbildungsinhalte und Gespräche erfolgt mittlerweile mit den eLogbüchern der Ärztekammern. Außerdem wird die Weiterbildung durch die neue (Muster‑)Weiterbildungsordnung kompetenzbasierter ausgerichtet.

Die strukturierte Erfassung von EPAs – „entrustable professional activities“: in sich geschlossenen, praktischen klinischen Tätigkeiten, welche mess- und beurteilbar sind – trägt zur dieser kompetenzbasierten Neuausrichtung der Weiterbildung bei. Somit kann der Lernfortschritt transparenter festgehalten werden [26, 27]. Im Pilotprojekt am Universitätsklinikum Hamburg-Eppendorf wurde ein digitales, EPA-basiertes Weiterbildungscurriculum, zusätzlich zum klinikinternen Lerncurriculum, entwickelt [28, 29]. Eine bundesweite Weiterentwicklung ist denkbar mit Anpassungsmöglichkeiten für jede Klinik. Außerdem ist eine Erweiterung dieses digitalen Tools durch die Erfassung der Anzahl an speziellen Maßnahmen (z. B. zentralvenöse Katheterisierungen) mit einer direkten Verknüpfung ins eLogbuch mit digitaler Unterschrift des Weiterbildungsbefugten denkbar.

### Integration von digitalem Wissen

Bei der Entscheidung, wo die Weiterbildung begonnen wird, stehen für viele frisch approbierte Kollegen neben dem klinischen Spektrum v. a. die Möglichkeiten in der Weiterbildung an oberster Stelle [30].

Insgesamt streben viele anästhesiologische Kliniken, so auch die Kliniken der Autoren, in der Weiterbildung einen mehrdimensionalen Ansatz an. Inhaltlich sind für die Weiterbildung der Ärzte in erster Linie neben dem Einrichtungsleitern selbst erfahrene Fach- und Oberärzte zuständig; zusätzlich liegt es aber an jedem Individuum selbst, sich auch außerhalb der Klinik kontinuierlich fortzubilden, um neue Inhalte zu erarbeiten und zu vertiefen [31]. Als dritte Ebene ermöglichen es aber gerade die neuen digitalen Tools, externe Fort- und Weiterbildungsinhalte in die abteilungsspezifischen Weiterbildungscurricula zu integrieren. Im nächsten Abschnitt geht es konkret um einfachere sowie komplexere digitale Inhalte, die in die institutionelle Weiterbildung integriert werden können.

#### Einsteigerinhalte

Allgemein sollten digitale Inhalte strukturiert in das Weiterbildungskonzept integriert werden. Wenn ein klinikinternes Lerncurriculum vorhanden ist, kann analysiert werden, welche Anteile durch Digitalisierung besser zugänglich gemacht werden können (Infobox 1). Beispielshalber ist eine Verknüpfung von Lernmodulen und Rotationsplan denkbar. Jedes Mal, wenn ein Arzt in Weiterbildung einen neuen Weiterbildungsbereich beginnt, werden die dazugehörigen digitalen Ressourcen zur Verfügung gestellt.

Diese gute Auswahl an Ressourcen für jeden Wissensstand, ausgesucht und bewertet durch die jeweiligen fachärztlichen und oberärztlichen Kollegen, sollte fester Bestandteil des Zugangs zu Wissen für alle Weiterbildungsinteressierte sein. Zu dieser Auswahl würden u. a. Podcasts, wissensvermittelnde Videos und andere digitale Inhalte sowie klassische Lehrveranstaltungen, Konferenzen und Literatur gehören; auch eine Liste von relevanten X‑ (ehem. Twitter‑)Hashtags oder Instagram Accounts ist nützlich. Diese Listen sollten regelmäßig aktualisiert werden und digital zur Verfügung stehen. Tab. 1 zeigt beispielhaft einige allgemeine digitalen Medien.

**Tab. 1:** Allgemeine digitale Tools mit Beispielen und Verlinkungen

Digitales Medium	Anbieter/Plattform	Links
	Beispiele/Accounts	
Lehrvideos	YouTube	https://www.youtube.com/
	Vimeo	https://vimeo.com/
Podcasts	*Anbieter*	
	Spotify	https://open.spotify.com/
	Apple Podcasts	https://www.apple.com/apple-podcasts/
	Google Podcasts	https://podcasts.google.com/
	Deezer, u. v. m.	https://www.deezer.com/
	*Beispiele*	
	YUAN – Young Urban Anesthesiologists	https://ains.umg.eu/studium-lehre/podcast/
	EmCrit	https://emcrit.org/category/emcrit/
	FOAMcast	https://foamcast.org/
	CODA Change	https://codachange.org/podcasts
Blogs und Webseiten	Nerdfallmedizin	https://nerdfallmedizin.blog/
	Life in the Fastlane	https://litfl.com/
	The Bottom Line	https://www.thebottomline.org.uk/
	pin-up-docs – don’t panic	https://pin-up-docs.de/
	dasfoam.org	https://dasfoam.org/
	Critical Care Reviews	https://criticalcarereviews.com/
	Anesthesia Guidebook	https://anesthesiaguidebook.com/
	OpenAnesthesia	https://www.openanesthesia.org/
	Deranged Physiology	https://derangedphysiology.com/
	Propofology.com	https://www.propofology.com/
	u. v. m.	–
Apps	AnSo	App im bevorzugten App Store suchen
	PediHelp	
	AINS	
	AMBOSS	
	EchoCalc	
	DGK-Leitlinien	
	eGENA	
	Instagram	@nysora.inc @rishimd @esicm_intensivecare u. v. m.
	u. v. m.	
	u. v. m.	–

**Abb. 3:**
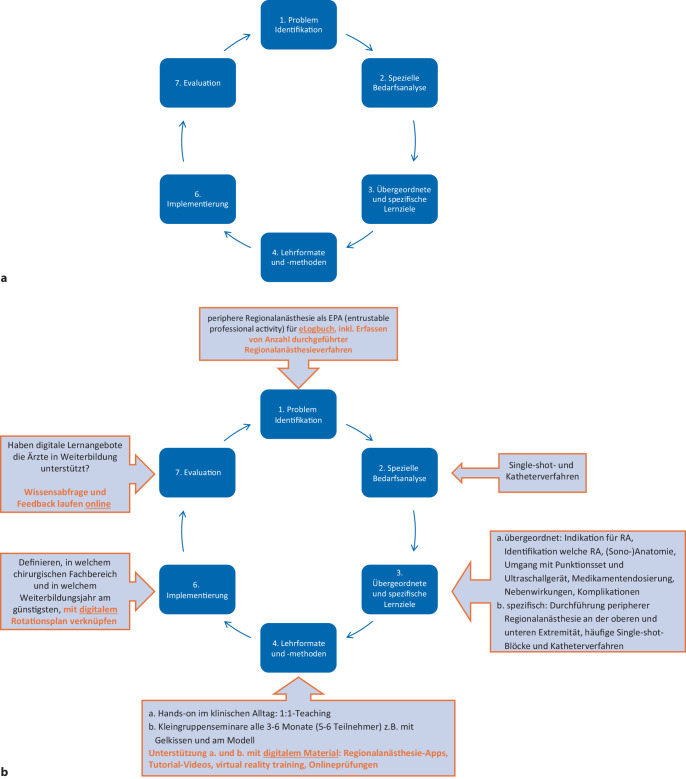
Kern-Zyklus. **a** Allgemein (nach Thomas et al. [32]), **b** für die Regionalanästhesie. *Orangefarben* Möglichkeiten der digitalen Verknüpfungen

Zur Integration von digitalen Inhalten können z. B. Kernzyklen als übergeordnete Struktur hilfreich sein. Der Kernzyklus (Abb. 3) ist der Goldstandard für die Entwicklung von medizinischen Curricula. Dieses Konzept wurde auch für die Online-Lehre weiterentwickelt [32, 33]. Zweifellos sind in vielen Curricula schon digitale Inhalte vertreten, aber durch eine geordnete Sammlung an Ressourcen und Quellen können diese immer wieder ergänzt werden.

Als Beispiel aus der Praxis nehmen wir hierfür wir die Regionalanästhesie als Teilkompetenz der Anästhesiologie (Abb. 3b), wie wir diese teilweise am Standort Rostock in das Weiterbildungscurriculum eingebettet haben. Die Integration von digitalen Inhalten zu diesem Thema kann an fast allen Schritten im Kernzyklus erfolgen.

Die Inhalte der Schritte sollten auch miteinander verknüpft sein, um den bestmöglichen Lernerfolg der Teilnehmenden zu ermöglichen. Es ist für die Autoren klar, dass Online-Angebote bei vielen EPAs und allgemeinen Lehrinhalten in allen Bereichen der AINS sowie Palliativmedizin das Lernen unterstützen sollten.

Neben den offiziellen Online-Ressourcen, die abteilungsweit empfohlen werden, entstehen automatisch unter den Kollegen auch immer informelle Sammlungen an digitalen Materialien. Eine Einbindung dieser informellen Sammlungen, neben den offiziellen Ressourcen, im Sinne eines offenen Systems, hat sich durchaus als vorteilhaft erwiesen: Die Begeisterung für das Lernen neuer Techniken wird unterstützt, und das mit einer oftmals beeindruckenden Dynamik: interessante Artikel, Blogs, Podcast-Folgen und Videos werden schnell geteilt. Aufgrund dieser Dynamik sind diese Ressourcen, d. h. nicht fachärztlich geprüft, auch eine wertvolle Quelle. Wichtig ist aber, den Unterschied zwischen ungeprüfter und geprüfter Ressource immer zu verdeutlichen: So muss von Anfang an jedem Berufseinsteiger, die alle im Gegensatz zu den Klinikleitungen „Digital Natives“ sind, nachdrücklich vermittelt werden, dass Inhalte immer kritisch zu evaluieren sind. Dieser Prüfung sowie fortgeschrittenen Inhalten widmen wir uns mit im nächsten Abschnitt.

#### Fortgeschrittene Inhalte: FOAM u. v. m.

Neben den oben besprochenen, prinzipiell in den Abteilungen generierten bzw. administrierten digitalen Möglichkeiten ist in den letzten Jahren ein großes Feld an sehr heterogenen Online-Lernangebote entstanden, welche unter dem Begriff „Free Open Access Meducation“, kurz „FOAM“, bekannt sind. Meducation ist eine Zusammensetzung aus „medical“ und „education.“ Das Prinzip von FOAM ist im modernen Weiterbildungskontext unseres Fachgebietes nicht mehr wegzudenken. Die oben beschriebenen allgemeinen digitalen Tools können nun genutzt werden, um FOAM-Inhalte strukturiert in Weiterbildungskonzepte zu integrieren.

“If you want to know how we practiced medicine 5 years ago, read a textbook. If you want to know how we practiced medicine 2 years ago, read a journal. If you want to know how we practice medicine now, go to a (good) conference. If you want to know how we will practice medicine in the future, listen in the hallways and use FOAM” [34].

Deutsch: „Wenn Du wissen willst, wie wir vor 5 Jahren Medizin gemacht haben, lies ein Lehrbuch. Wenn Du wissen willst, wie wir vor 2 Jahren Medizin gemacht haben, lies eine Fachzeitschrift. Wenn Du wissen willst, wie wir jetzt Medizin machen, geh auf eine (gute) Konferenz. Wenn Du wissen willst, wie wir in Zukunft Medizin machen werden, hör auf den Fluren zu und nutze FOAM.“

Dieses Zitat stammt von Dr. Joseph Lex, Professor der Notfallmedizin an der Temple University, Philadelphia, USA. Er war einer der ersten Experten, der kostenlose Vorträge über Notfallmedizin online gestellt hat [35]. Es zeigt anschaulich, wie lange Wissensvermittlung – „knowledge translation“ – bis zur praktischen Anwendung ungefähr dauert.

FOAM ist inzwischen fast synonym zu den neuen, digitalen Weiterbildungsmöglichkeiten in der Medizin. Ursprünglich sei FOAM 2012 in einem Pub in Dublin entstanden: Dr. Mike Cadogan, Notfallmediziner aus Australien, sollte auf einer Konferenz einen Vortrag über die Rolle der sozialen Medien bei der medizinischen Bildung halten. So soll ihm beim Blick auf den Schaum (engl. „foam“) in seinem Bierglas die Idee für diesen Oberbegriff gekommen sein. FOAM schließt die kostenlosen Medien Blogs, Podcasts und Webinars sowie die Nutzung von Social Media zur Verteilung von Medizinwissen ein [36].

Für den Erfolg von FOAM war die zunehmende Nutzung der sozialen Medien i. Allg. sowie durch das medizinische Personal als schnelle und offen zugängliche Kommunikationsplattform essenziell. Die Anzahl der Social-Media-Nutzer soll 2027 auf ca. 6 Mrd. wachsen [37]: Junge Ärzte verbringen einen großen Teil ihrer Freizeit damit, im Internet zu surfen, zu lernen, Informationen auszutauschen und allgemein zu kommunizieren [38]. Laut 2 irischen und australischen Studien nutzen über 90 % der präklinischen und klinischen Medizinstudierenden schon wöchentlich Online-FOAM-Angebote [39, 40]. Insbesondere während der Coronapandemie wurden Social-Media-Plattformen noch ausgiebiger für Wissensaneignung und -dissemination genutzt [41, 42].

Bereits 2002 wurde das Potenzial erkannt, über das Internet die medizinische Ausbildung zu verbessern. In den darauffolgenden Jahren folgten mehrere Fachkonferenzen, die sich zunehmender Beliebtheit erfreuten. Als große Vorbildkonferenz ist SMACC zu nennen: „Social Media and Critical Care“; obwohl diese Konferenz in dieser Form nicht mehr existiert (spätere Form: CODA Change), hat sich daraus eine Bewegung, die sich der frei zugänglichen Wissenschaftskommunikation für medizinisches Personal gewidmet hat, entwickelt. Ziel ist es, Informationen und Ausbildungsmaterial, ansprechend gestaltet, online zur Verfügung zu stellen; durch den freien Zugang zu verschiedenen Medientypen – Videos, Blogeinträge, Podcasts – ermöglicht dies das asynchrone Lernen nach der individuellen Präferenz des Einzelnen. Hiermit wurde FOAM als „dritte Welle“ mehr Struktur verliehen. Laut Chan et. al sind wir mittlerweile in der vierten Welle der Entwicklung von FOAM angekommen, bei der sich die Endverbraucher mitengagieren und mitwirken und sich so sowohl als Lernende als auch Lehrende engagieren [36].

Wichtig ist jedoch auch hier, kritisch potenzielle Limitationen und auch Gefahren nicht zu ignorieren: Wenn hochmotivierte Mediziner selbstbestimmt Inhalte verbreiten, entstehen sehr vielschichtige Angebote, sodass für jeden interessante Inhalte verfügbar sind. Die freie Auswahl der produzierten und verbreiteten Inhalte hat jedoch auch den Nachteil, dass diese nicht zwangsläufig repräsentativ für die Inhalte des jeweiligen Faches sind. So lässt sich beobachten, dass anfangs gerade besonders glamouröse und extreme Inhalte überrepräsentiert waren [43]. Ähnlich wie in anderen Sozialen Medien haben sich auch in Bezug auf FOAM inzwischen „Stars“/Influencer der Szene gebildet, sodass hier auch die Gefahr einer „eminence-based medicine“ besteht [44].

Ein weiterer Kritikpunkt sowie gleichzeitig auch eine Stärke von FOAM ist der Veröffentlichungsprozess [45–47]. Durch die Offenheit des Internets kann jede Person FOAM-Inhalte produzieren. Dabei findet kein klassischer Peer Review statt, sodass jede Publikation mit einer gewissen Skepsis begutachtet werden muss. Durch die öffentliche Diskussion und die schnelle und digitale Korrespondenz findet allerdings eine Art Post-Publication Review statt. Es ist ein integraler Teil der FOAM-Szene, dass eine rege, konstruktive und v. a. für alle offene Diskussion der Inhalte stattfindet. So können über die Schwarmintelligenz inhaltliche Fehler schnell korrigiert werden [9, 48]. Außerdem gilt es, allgemeine „good practices“ sowie Netiquette (angemessenes und achtendes Benehmen in der technischen Kommunikation) einzuhalten [49].

Als Beispiel für einen schnellen Veröffentlichungsprozess ist eine Stellungnahme der American Society of Regional Anesthesia and Pain Medicine/European Society of Regional Anesthesia and Pain Therapy zur Therapie chronischer Schmerzen während der Coronapandemie zu nennen. Nach einem Tweet (Beitrag auf der ehem. Plattform Twitter) am 15.03.2020 entstand nach Diskussion zwischen internationalen Experten, zwei Wochen später (!), eine Handlungsleitlinie, welche offiziell publiziert wurde [50]. Umgekehrt ist auch die schnellere Wissensumsetzung („translation of knowledge“) möglich [6, 51, 52]. 2016, innerhalb von wenigen Tagen, hatten mehrere FOAM-Kanäle einen klassischen Journal-Artikel zu den neuen Sepsisleitlinien (*JAMA*, Sepsis-3) geteilt. Hieraus entstanden z. B. mehrere Podcastfolgen des Formats „EMCrit“, bei dem Dr. Weingard Interviews mit den Autoren führte bzw. Fragen aus der Sepsis-Experten-Community beantwortete [44].

Zwar ist das Wissen um die Schwarmintelligenz hilfreich, jedoch gibt es keine Hilfe in der persönlichen Qualitätsbeurteilung, wenn es um neue FOAM-Inhalte geht. Durch die zunehmende Menge an Inhalten, die sich selbst als FOAM bezeichnen, erscheint es sinnvoll, auch hier Qualitätsmarker zu etablieren [44]. Grundsätzlich muss an dieser Stelle zwischen Websites und einzelnen Inhalten unterschieden werden. Ein Beispielmarker für Websites ist der sog. Social Media Index (Smi); er basiert auf mathematischen Berechnungen, die Aufrufe der entsprechenden Website sowie Präsenz bei Social Media einschließen. Er soll ein Pendant zum klassischen Impact Factor darstellen. Es gibt Arbeiten, die eine Korrelation des Smi zu Impact und Qualität stützen [53, 54]. Der Smi kann auch eine Möglichkeit sein, um neuen Nutzer eine Einführung in gute Medien zu bieten. Gleichwohl wird hier nur die Qualität der Website i. Allg. bewertet. Dies schließt nicht aus, dass es Artikel oder Inhalte geben kann, die nicht den üblichen Qualitätskriterien entsprechen.

Für einzelne Inhalte haben sich zwei Methoden etabliert, die letztendlich auch für die allgemeine Bewertung von Inhalten im Internet gelten: Hierbei handelt es sich um den rMETRIQ Score und den AliEM Air Score [54–56]. Diese beiden Scoring-Systeme benötigen ein wenig Zeit zur Einarbeitung und wirken erstmal etwas sperrig.

Angelehnt an die Inhalte dieser beiden Scoring-Systeme wollen die Autoren an dieser Stelle einen Vorschlag für Qualitätskriterien für FOAM-Inhalte darstellen. Diese sind auch hilfreich für die Integration von digitalen Inhalten in die Weiterbildungsinhalte als Teil eines übergeordnetes Digital-Media-Konzepts (Infobox 6).

##### Infobox 6: Qualitätskriterien FOAM-Inhalte

- Sind Klarnamen als Autoren verwendet?
- Sind potenzielle Interessenkonflikte der Autoren adressiert?
- Sind Rücklaufkanäle zugelassen, d. h., können Diskussionen nachverfolgt werden?
- Sind adäquate Quellenangaben vorhanden?
- Entsprechen die Formatierung und der Ausdruck gängigen Standards für wissenschaftliche Texte?

Insgesamt haben FOAM-Inhalte, die vor einigen Jahren eher nur Zusatzquellen für Wissensaneignung waren, an Wichtigkeit enorm zugenommen [30, 51, 57]. Diese können sinnvoll in jedes Aus- und Weiterbildungskonzept einer medizinischen Abteilung aufgenommen werden und sollten als ein „*und*“ und kein „*oder*“ bei der Auswahl an Quellen verstanden werden.

Nebenbei bemerkt wird die Bewegung der „*free* open access medical education“ zunehmend kommerzialisiert; der Weg geht weg von einer „kostenfreien“ Open-Access-Lösung hin zu privatwirtschaftlichen Geschäftsmodellen. Dies sollte uns nach Meinung der Autoren gerade auch auf Fachgesellschafts- und Verbandsebene wie auch aus den akademischen Einrichtungen heraus motivieren, Angebote zu generieren, die im Sinne der Web‑2.0‑Bewegung barrierefrei für die Aus‑, Fort- und Weiterbildung zur Verfügung stehen [57].

Abgesehen von der Generierung von neuem Wissen ist auch die Wissenschaftskommunikation mit dem Ziel des Screenings von Quellen und der Aufbereitung und Dissemination von Wissen ein entscheidendes Stichwort. Kontinuierliche medizinische Fortbildung im Sinne des lebenslangen Lernens wird insofern erleichtert, als dass es so möglich ist, individuell passende Angebote für alle Lerntypen zu finden oder zu generieren. Alle Kollegen können hiervon profitieren. Auf einer nationalen Ebene können sich die Autoren ein Netzwerk an medieninteressierten Einrichtungen vorstellen, die gemeinsam ein Digital-Media-Siegel, welches für die Qualität der FOAM-Inhalte bürgt, erarbeiten und dann verteilen. Eine ähnliche Idee steckt hinter dasFoam Think Tank, wo FOAM-interessierte Individuen sich gemeinsam mit FOAM-Inhalten beschäftigen.

## Fazit für die Praxis und Blick in die Zukunft

Jeder Kollege, egal ob lang berufserfahren und von der konventionellen, analogen Lehre geprägt oder der „Digital Native“, frisch aus dem sich digitalisierenden Medizinstudium, sollte sich mit dem Thema digitale Werkzeuge auseinandersetzen. Es gibt viele Angebote für jeden Lerntyp und für jedes Zeitbudget. Für den optimalen Einsatz und v. a. auch für die sinnvolle Zukunftsgestaltung der digitalen Weiterbildung ist neben individueller Eigenmotivation jedes Einzelnen auch viel Verantwortung und Weitsicht seitens der Einrichtungsleitungen erforderlich. Das Zusammenspiel von konkretem Digital-Media-Konzept einer Abteilung und Eigeninitiative birgt das Potenzial, die Weiterbildung inhaltlich zu verbessern, aber v. a. auch den Nachwuchs für unser Fach zu begeistern und ihn auch dort zu halten. So kann eine Podcast-Folge z. B. als Basis genutzt werden, medizinische Inhalte kritisch im eigenen Kontext zu diskutieren – und das mit Enthusiasmus und Leidenschaft, weil es auch den Zeitgeist trifft. Gleichzeitig muss darauf geachtet werden, dass, obwohl FOAM und persönliche Lernnetzwerke viel Positives zum Lernverhalten beitragen können, die verwendeten Inhalte vertrauenswürdig im Sinne von medizinischer und wissenschaftlicher Konsistenz sind. Digitale Tools bieten so auch eine sinnvolle Möglichkeit der Annäherung von „Work and Life“ und deren viel beschworener, schwieriger Balance.

Erst 2017 wurde über die digitalen Medien gesagt, dass sie im Medizinstudium noch kein integraler und flächendeckender Bestandteil der Lehre seien [58]. Dies hat sich in den letzten 7 Jahren, und insbesondere während der Coronapandemie, drastisch geändert; digitale Tools sind gekommen, um zu bleiben. Jedes Weiterbildungscurriculum kann davon profitieren [59]. Es ist essenziell, den existierenden wie auch den noch kommenden digitalen Tools und Online-Ressourcen offen gegenüberzustehen und zu analysieren, ob und wie sie zum Fach, zur Weiterbildung, und zu den Weiterbildungsstätten passen. Leider sind wir in Deutschland im internationalen Vergleich in Bezug auf die digitale Infrastruktur i. Allg. sowie im Gesundheitswesen nicht die Benchmark [60]. Im Positionspapier des Bündnis Junge Ärzte wurde 2020 zusammenfassend gefordert, um so stringenter mit den neuen digitalen Entwicklungen mitzugehen [61]. Auf dem Deutschen Ärztetag 2017 wurde bereits diskutiert und essenziell festgehalten, dass die Anpassung an neue digitale Medien zur Weiterentwicklung von Wissenschaft, Ausbildung sowie Patientenversorgung von den Ärzten vorangetragen werden muss [62], bevor dies aus primär kommerzieller Sicht seitens der Industrie geschieht [60]. Die Digitalisierung, inkl. künstlicher Intelligenz, wird das Gesundheitswesen maßgeblich in der Zukunft mitgestalten [63].

Einen unmittelbaren, konkreten Schritt, um den Umgang mit digitalen Medien in der eigenen Abteilung zu begleiten, stellt das Ausarbeiten eines strukturierten Digital-Media-Konzepts und die Umsetzung erster Schritte dar. Allein schon die Bereitstellung asynchroner Fortbildungsangebote kann viele Kollegen signifikant unterstützen. Insbesondere Eltern, die andere Bedürfnisse im Gegensatz zu kinderlosen Kollegen haben [64, 65], nehmen dies gut an. Das Streben, eine persönliche Work-Life-Balance zu finden, ist in der Generation Y und Z sehr hoch [66]. Die proaktive, konsequente Integration neuer Medienformate mag auch hier eine Brücke, die signifikant zur Attraktivität unseres Faches beitragen kann, bauen.

Digitale Medien zu nutzen, um eine positive Lern- und Arbeitsatmosphäre für jeden ärztlichen Kollegen in ganz unterschiedlichen Phasen des Arbeitslebens zu schaffen, erscheint möglich und es ist wichtig, diesen Brückenschlag weiter zu unterstützen. Von einem Lernvideo als Ausgangspunkt für Diskussionen über Weiterbildungsangebote im „Flipped-Classroom-Konzept“ und den strukturierten Einsatz von FOAM-Inhalten bis hin zur Augmented-Reality Technology im Simulationstraining – digitale Möglichkeiten und Methoden sind bereits eine substanzielle Unterstützung zum lebenslangen Lernen in der Anästhesiologie, Intensivmedizin, Notfallmedizin und Schmerztherapie und werden zukünftig noch viel größeren Raum einnehmen.

## Fazit für die Praxis

- Digital abrufbare Angebote in der Fort- und Weiterbildung der Anästhesiologie, Intensivtherapie, Schmerz‑, Notfall- und Palliativmedizin sind vielfältig und heterogen.
- Ein Digital-Media-Konzept, welches auf die individuellen Bedürfnisse einer Abteilung maßgeschneidert wird, ist empfehlenswert.
- Online-Inhalte sollten strukturiert in die Praxis der Fort- und Weiterbildung eingebunden sowie traditionelle Lehrinhalte online zugänglich gemacht werden. Hierzu zählen z. B. die Nutzung von Apps, das Erstellen von einem Verzeichnis mit geprüften Online-Quellen für verschiedene Weiterbildungsthemen und/oder das Bereitstellen von aufgezeichneten Fortbildungsveranstaltungen.
- Zu der Entwicklung eines Digital-Media-Konzepts gehört u. a., Ziele und Ressourcen der Abteilung zu erfassen, Verantwortliche zu benennen, Qualitätskriterien zu festlegen und Informationen zu vernetzen sowie aktuell zu halten.
- Durch die Integration von digitalen Lehrangeboten in den Wissenserwerb wird auf verschiedene Lerntypen und Dienstmodelle einer Abteilung eingegangen.
- Digital Tools bereichern und unterstützen jeden Kollegen auf dem Weg des lebenslangen Lernens.
